# Managing social networking stress: the role of self-management in reducing social media exhaustion and improving higher education employee performance

**DOI:** 10.3389/fpsyg.2023.1254707

**Published:** 2023-12-29

**Authors:** Wajiha Moughal, Shahrina Md. Nordin, Rohani Bt Salleh, Haider Ali Abbasi

**Affiliations:** Center of Social Innovation, Department of Management and Humanities, Universiti Teknologi PETRONAS, Seri Iskandar, Malaysia

**Keywords:** social networking sites, social media exhaustion, self-management, job performance, stressor-strain-outcome theory, Smart PLS, academic staff

## Abstract

This study investigates the significance of self-management in academic staff stress management related to social networking sites (SNS). It emphasizes particularly on reducing social media exhaustion and increasing job effectiveness. The research applies the stressor-strain-outcome theory and the Smart PLS (partial least squares) analytical approach to examine data from 391 respondents. The study’s goal is to provide empirical data on the efficacy of self-control management in reducing SNS stress and its effects on academic staff’s psychological wellbeing and job performance. Data is collected by survey using online email platforms among academic employees, and the collected data is examined utilizing the Smart PLS approach. This approach allows for an investigation of the proposed links and their statistical importance. This research’s ramifications are important for academic institutions since its results can help academic personnel effectively cope with SNS-related stress. Academic employees can better limit their SNS usage and avoid social media tiredness by promoting self-control management practices. As a result, academic employees’ job performance and overall wellbeing may increase. The study’s findings help to comprehend how self-management might reduce SNS stress and improve staff performance in the academic sector.

## 1 Introduction

Social networking sites (SNSs) like Instagram, Facebook, and Twitter have revolutionized how people interact and communicate within enterprises. SNSs are being used more widely and are becoming more well-liked (Dimock, 2019). However, the enticing features of SNSs can cause users to check their accounts frequently and relentlessly (Cao and Sun, 2018). The use of SNSs in a variety of ways, such as likes, using chat platforms, and status updates, may ultimately set off indicators and cluster toward behavior as cognitive and emotional reactions, which is likely to make it challenging to manage how one uses social media (Barrot and Acomular, 2022). In fact, excessive usage of social media can lead to signs of dependency emerging, which may have detrimental psychological and behavioral effects of stress. Numerous studies suggested that SNSs users have inadequate manipulation over their usage, which can interfere with daily activities in the home, workplace, and school (Turel et al., 2018). Employees usually use SNSs to the point where they might log in for a long time and get distracted (Boyd and Ellison, 2007; Ellison et al., 2014; Vishwanath, 2015). SNSs can improve work-related outcomes by connecting people and giving them the accessibility to services at work needed for improved performance (Leidner et al., 2010; Wu, 2013; Ellison et al., 2014). But frequent use of SNSs could have detrimental effects like stress, addiction, exhaustion, diversion, a decrease in pleasant emotions, poor performance, and bad health (Bevan et al., 2014; Fox and Moreland, 2015), which reduce the output of employee job performance.

Because of this, there has been an upsurge in research related to the sources and effects of using SNSs (Milošević-Ðorđević and Žeželj, 2014; Tang et al., 2016; Turel et al., 2018; Elsayed, 2021). Despite growing interest in SNSs use, little is known about how it affects results in both personal and professional spheres. Additionally, a theoretical framework that explicitly explains how SNSs usage distracts employees, could affect various contexts and exhaust social media usage which reduces employee job performance which is lacking in the present research related to SNSs. Such type of research is needed which can use SNSs where self-management can reduce social media exhaustion and improve employee job performance which tends to be challenging in a particular condition. These constraints will be addressed in the present study by fusing SNSs usage with both private and professional repercussions through stressors-strain-outcome theory. In SSO theory explains the framework of the study that Excessive social at work, excessive hedonic use at work, excessive cognitive use at work (stressors) that make working situations uncomfortable for the employees and lead to social media exhaustion (strain) and reduce their job performance (outcome). The users spend a lot of time on the internet and social networking sites that negatively affect employee job performance.

Our theoretical approach is based on Bandura’s reciprocal determinism notion in information and communications technology (ICT) (Bandura, 1986), which holds that an individual’s behavior can alter how the environment (both at work and in their personal lives) is viewed by them and how they interact with it. We focus on a selection of significant personal and professional effects of SNSs usage in this study. This study pays close attention to both individual aspects, such as healthy feelings and pleasant emotions, and professional factors, such as social media exhaustion, self-management, and job performance. The term “positive impulses” refers to the psychological experiences that enhance and strengthen one’s “persistent personal resources” and enable behaviors that lead to favorable results in both one’s personal and professional life (Fredrickson, 1998). Prior research has examined the importance of work environment elements, including job distraction and performance, and their effects (Janssen and Van Yperen, 2004; Liang et al., 2011; Zwarun and Hall, 2014; Tarafdar et al., 2020b), but the effect of self-management to reduce social media exhaustion has not been investigated to improve the performance. This study intends to examine the effect of self-control management in reducing social media exhaustion caused by social networking sites stressors and improves employee performance. The current will be helpful for the institutions to devise strategies based on self-control management to improve employee performance.

This study has several significant theoretical and practical consequences. By providing a theoretic justification and practical evidence for the effects of SNSs practice on employee performance in professional contexts, this research enlarges the growing body of literature concerning frequent SNSs usage in the workplace can be reduced by self-management to improve overall performance. In practical terms, this research contributes to the direction of technological resources through employee perspective providing a better knowledge of self-management among employees to reduce SNSs exhaustion, which may lead to the provision of recommendations that can help private and public organizations to mitigate the usage of SNSs in the workplace.

Stress at work could be triggered by many working aspects such as organization management, workload, excessive use of SNSs, structural and organizational climate, job characteristics and many more. A finding of a research done in one public university revealed that employees added stress when they felt not appreciated by the bosses, received orders outside office hours and excessive use of SNSs during work, getting pressure from the admins to achieve the Key Indicator Performance (KPI) of the departments (Isa and Kadir Shahar, 2021).

This study aims at deepening our understanding on how different technology-related stressors indirectly create negative outcomes. We specifically focus on enhancing the job performance as outcome because it has been demonstrated that it is a key detrimental state for employee exhaustion and relationship with the organization. Focusing on this outcome, we propose a model that explains how adaptive and maladaptive coping strategies influence it, and how they are indirectly informed by key techno-stress creators, namely excessive social use at work, excessive hedonic use at work and excessive cognitive use at work.

The structure of this paper is as follows. The pertinent literature is briefly reviewed in the part that follows, with an emphasis on SNSs. The literature review will be addressed and followed by wrapping up with testable theoretical hypotheses. The outcomes of the structural equation modeling analysis are then presented, trailed back by a description of the research process. This study discussed the study’s ramifications, recommendations for additional research, study limitations, and findings.

## 2 Context of the study

In Malaysia, academic and non-academic staff have been under pressure over the years to meet the increasing demands to be internationally recognized academically and in the field of research, generate income for the university, and they were expected to meet the performance set by the university (Mukosolu et al., 2015). Job-related stress among academic university staff has more attention among researchers than other occupational groups in the UK (e.g., Kinman and Jones, 2003; Tytherleigh et al., 2005). Conversely, this research is examining the impact of SNSs stressors on the productivity of university academic staff. As academic staff are under stress as they need to respond to the global demands, their responsibilities are no longer confined within the boundaries of teaching and learning. As the status of higher education is changing according to the needs of globalization, it goes parallel with the responsibilities of academic staff (Sidek et al., 2012).

## 3 Literature review

### 3.1 Excessive use of SNSs

Social networking sites are frequently accessed by employees as a distraction from challenging or time-consuming work situations or to put off finishing responsibilities. Much research suggests compulsive Internet use, with some users becoming overly reliant on web-based communication applications (SNSs). This could lead to a variety of impairments, such as psychological, social, and professional impairments (Young, 1996; Orzack and Orzack, 1999; Griffiths, 2000; Meerkerk et al., 2009; Blackwell et al., 2017; Carbonell and Panova, 2017).

### 3.2 Three dimensions of excessiveSNS use

According to Yu et al. (2018) and Cao et al. (2019) research, when people use social media excessively, they believe that much time and effort is being wasted while at work. There are three types of excessive social media use at work: excessive hedonic, excessive social, and excessive cognitive.

### 3.3 Excessive social use at work

In order to use SNSs for social purposes, users must engage in two-way communication with friends, family, and other network users. This communication helps users establish and sustain social relationships (Luqman et al., 2017). Excessive social use at work is defined as putting too much time and effort into using social media to establish and maintain professional contacts (Ali-Hassan et al., 2015). But overuse of social media can encourage users, which can lead to compulsive behaviors like constant checking (Yu et al., 2018; Abbasi et al., 2021a). Numerous pieces of data show that SNS social users lack effective self-control, which interferes with daily activities in the home, workplace, schools, and businesses (Cao and Sun, 2018).

### 3.4 Excessive hedonic use at work

The hedonistic usage of social media is predicated on pleasure and fun, similar to that which is had while playing games and watching entertainment (Zheng and Lee, 2016; Luqman et al., 2017). When someone uses social networking sites (SNSs) excessively to relax, escape from, or amuse themselves while at work, it’s referred to as “extreme hedonic use at work” (Ali-Hassan et al., 2015). Extreme SNSs users use SNSs applications, play games, or watch movies for a sizable portion of their workday (Carbonell and Panova, 2017). Users of SNSs frequently use the platform to fulfill their personal demands rather than complete their work obligations, which has a detrimental impact on how successfully they accomplish their employment. Numerous studies have found a connection between the use of SNSs and poor student and employee performance in the classroom (Benson et al., 2019; Abbasi et al., 2021b). Additionally, hedonistic use of SNSs includes compulsively rewarding behavior, which raises the danger of long-term usage and the urge to continue using it (Luqman et al., 2017).

### 3.5 Excessive cognitive use at work

The final aspect of using social networking sites, cognitive use, is involved with creating content to share and accessing content created by others, such as reviews, tales, ratings, images, and videos (Cao and Yu, 2019). When people “produce and exchange content, ideas, and opinions; build habits; participate in debates; see photographs; and consume content posted by other users,” they are using social networking sites (SNS) cognitively (Papacharissi and Gibson, 2011; Luqman et al., 2017). According to Deryakulu and Ursavaş (2014), using social media (SNS) extensively while at work to create and distribute user-generated content is referred to as excessive cognitive use. Thanks to information technology, people now have greater access to information and communication than ever before, however, one study found that the harmful impacts of “over information” have also attracted increased attention from researchers (Luqman et al., 2017; Abbasi et al., 2021c). Because of this, the employee’s capacity is impaired by excessive cognitive use, which also affects their ability to perform their daily activities at work. Due to being distracted, employees could perform worse at work (Hou et al., 2017).

### 3.6 The influence of excessive social use at work on social media exhaustion

Among other technologies, social media platforms like instant messaging (IM) can be used to improve collaboration within organizations (Davison et al., 2014). Once connected via social media, workers prefer to put everything on hold and respond to contact requests instantly (Davison et al., 2014). It normally takes a few minutes to return to previous job duties after processing a communication break that was instigated by another party (Nisar and Whitehead, 2016). Given the limitations of the human brain, unexpected encounters may lead to someone’s attention being distracted and their cognitive load increasing. People who stay in touch would therefore find it challenging to focus on their work, which would lead to them becoming weary and experiencing weary when using social media.

The overuse of social media at work, which has been linked to stress and social media exhaustion (Ayyagari, 2012; Sullivan and Koh, 2019), is the subject of this study. If someone uses social media excessively to establish and maintain business relationships, they could feel out of control (Ali-Hassan et al., 2015; Abbasi et al., 2022). Their mental health may be negatively impacted by these circumstances, which may ultimately result in anxiety and exhaustion (Ragu-Nathan et al., 2008; Lee S. L. et al., 2016). According to Cao and Sun (2018) and Moqbel and Kock (2018) compulsive monitoring behavior brought on by excessive sharing on social networking sites (SNS) can be exhausting and readily stopped.

Based on the theory and previous research above, it can be derived as follows:

H1: Excessive social use at work has a significant effect on social media exhaustion.

### 3.7 The influence of excessive hedonic use at work on social media exhaustion

According to Luqman et al. (2017), social media is about having fun and enjoying oneself, especially through games and entertainment. To pass the time while viewing films and playing games, there are a variety of apps accessible (Carbonell and Panova, 2017). When participating in social media, staff members typically put everything on hold to reply to requests (Eliyana et al., 2020).

Employees may feel emotions like curiosity, happiness, and enjoyment when they use the technology. As a result, it would be challenging for them to concentrate on their activity while engaged in constant contact; also, they would feel antsy and exhausted while using social media.

Based on the theory and previous research above, it can be derived as follows:

H2: Excessive hedonic use has a significant effect on social media exhaustion.

### 3.8 The influence of excessive cognitive use at work on social media exhaustion

Excessive cognitive use at work focuses on producing, disseminating, and accessing content that has already been produced by others. Examples of this content include opinions, tales, ratings, photos, and videos (Cao and Yu, 2019). Employees are diverted from their tasks when using SNSs at work, though. Employees who have self-awareness and self-management skills can still offset the negative impacts of excessive SNSs usage and cause social media tiredness.

The last aspect of social media use, known as cognitive use, involves producing, sharing, and accessing original content. This content includes, among other things, articles, photographs, videos, ratings, and opinions (Ali-Hassan et al., 2015). In order to influence task performance, employees typically use this function to collect and disseminate information. When they invest a lot of time learning and gathering information during working hours, employees are exposed to more extensive and in-depth information than is necessary (Landers and Schmidt, 2016). A person may experience information overload and be unable to fulfill their regular work commitments when they are unable to process a large volume of information (Eliyana et al., 2020). According to studies, a person’s ability to solve problems and make judgments may be impacted, reducing work productivity (Bawden and Robinson, 2009; Cao and Yu, 2019; Moughal et al., 2023a).

Employees who often access and exchange information via social media at work may experience information overload as a result of a high information load and cognitive process limitations (Roetzel, 2019). Due to the limitations of the human intellect, cognitive load occurs when employees are given more knowledge than they have the time or ability to digest and use (Eliyana et al., 2020). They could make you feel powerless. Technology weariness may result from such an incident having a negative impact on their mental health (Lee A. R. et al., 2016; Fu et al., 2020). Therefore, using social media at work in an excessively cognitive manner can lead to stressed-out employees.

Thus, we propose the following hypothesis:

H3. Excessive cognitive use has a significant effect on social media exhaustion.

### 3.9 The influence of social media exhaustion on job performance

Social media can become saturated if it is used excessively. A person’s emotional reaction to difficult events is referred to as exhaustion. When under difficult and prolonged conditions, exhaustion is the loss of mental energy (Kamal et al., 2020). Up to one-third of all online time is spent on social networking sites (SNSs), and many users claim to feel worn out as a result. Social media tiredness is a relatively new concept that deserves further study, according to some (Lee S. L. et al., 2016; Cao et al., 2019). According to Moqbel and Kock (2018), Social networking users (SNSs), for instance, may submit friend invitations and post about their daily life as the first source of it. Secondly, there is business interaction, including posting about the goods and services a company provides. The use of interfaces or the addition of new features comes third. Social media exhaustion has been brought on by the rising use of social media at work. This highlights the exhaustion that comes with utilizing technology.

Due to pressure to spend excessive amounts of time on social networking, employees may feel exhausted and stressed out (Zivnuska et al., 2019; Moughal et al., 2023b). They will thus perform less well at work (Fu et al., 2020; Ngien and Jiang, 2022). In light of the aforementioned theory and study, we can draw the following conclusions:

H4: Social media exhaustion has a significant effect on job performance.

### 3.10 The influence of self-control management on job performance

Self-control management is defined as the process of having higher control over one’s self while doing work (Wilson et al., 2018). Mezo (2009) identified three key elements of employee self-control management.

1. Self-monitoring
2. Self-evaluation
3. Self-reinforcement

The process of exercising greater self-control while working is known as self-control management (Wilson et al., 2018). In the current dynamic environment, self-management has evolved into a crucial quality of successful employees, according to Allegrante et al. (2019). Employees who are managed with self-control remain focused on their work and are able to carry out their responsibilities more effectively (Moore and Brown, 2019). Self-management is essentially keeping track of one’s behavior while one tries to complete a task and avoiding becoming sidetracked by other things like excessive SNS use or other distractions (Wheelock et al., 2015). Typically, a self-managed person makes plans for themselves and works hard to fulfill those objectives (Javed et al., 2019; Majid et al., 2019). Self-control management practices are helpful in enhancing employees’ intended behavior at workplace and managing undesired behavior, which might effect from impulses, ingrained habits, and behavior picked up through upbringing. Consequently, implementing self-management strategies at work can help organizations deal with numerous problems they currently confront (Javed et al., 2023). Employee job performance is positively impacted by internalizing the organization’s ideals through self-control management.

In essence, an employee’s capacity for self-management determines how well they perform (Javed et al., 2019; Moore and Brown, 2019). Self-management will be used as a moderating variable between SNSs stressors and employees’ performance based on the hypothesis that self-managed employees perform better. It is likely to moderate the association (Javed et al., 2019).

H5: Self-control management moderates the relationship between social media exhaustion and job performance.

### 3.11 Job performance

Many various terms are used to indicate how successfully someone does the work. The ability to accomplish work-related tasks is referred to as one’s job performance (Yu et al., 2022). This displays how adeptly an individual can fulfill the demands of their employment. A definition of “job performance” also includes “conduct or behavior that promotes organizational goals” (Ali-Hassan et al., 2015). The work product itself is essentially what the employees produce. Enhancing performance for both individuals and organizations is the main goal of initiatives to improve organizational performance (Moqbel et al., 2013).

According to Eliyana et al. (2020), performance refers to the results attained by an individual or group within a company. Performance in general refers to what employees do, and performance in particular refers to the quantity and quality of work that an employee completes by the responsibilities that have been assigned to him or her. Performance is often measured and associated with the accomplishment of certain company goals during those times. According to Darma and Supriyanto (2017) and Eliyana et al. (2020), job performance is a true behavior that everyone demonstrates as work performance created by employees in accordance with their position within the firm.

### 3.12 Stressor–strain–outcome (SSO) theory

Koeske and Koeske (1993)’s explication of the SSO model clarifies the connection between stressors, strains, and outcomes. According to this theoretical framework, a stressor has an indirect impact on the result, with stress being mediated through strain as social media exhaustion and typically producing unfavorable results (reducing employee performance) (Koeske and Koeske, 1993). The SSO theoretical framework is employed in the present research to clarify how excessive social media use, cognitive use and hedonic use at work lead to stress (Koeske and Koeske, 1993). Excessive social, hedonic, and cognitive usage in the workplace, excessive communication, and information overload are all elements that make working circumstances uncomfortable for employees and reduce their productivity. This study thus adds more information to the stressor-strain-outcome paradigm. This strategy is ideal for understanding the SNS stressors, looking into excessive social media use at work and its negative impacts, and looking into how social media exhaustion causes distraction and affects employees’ job performance.

**FIGURE 1:**
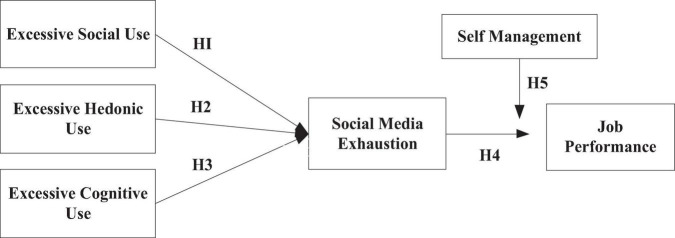
Conceptual framework.

This idea holds that the two fundamental elements of the stress phenomenon are stressors and strain. Stressors are external events that cause humans to experience strain, which is a psychological response. Stress is the result of an imbalance between a person’s true motivations, skills, and the need to maintain their surroundings. It includes unpleasant emotions including anxiety, fear, aggravation, pressure, and melancholy. The psychological stressors associated with technological stress that are most frequently studied in social media research are exhaustion (Lee and Lee, 2018), job fatigue (Meyer et al., 2015), and task distractions (Javed et al., 2019). Stress can also have a negative impact on an employee’s effectiveness at work. However, the link between stress and work performance is not fully explored from the standpoint of SNS stressors and the mediating effect of social media exhaustion to reduce performance. The performance of the company as a whole as well as that of the employee may be directly impacted by this. This can be a crucial sign to gauge how successful it was. Therefore, the stressor-strain-outcome model, which considers the user’s experience, is used in this study along with the mediating effect of social media exhaustion and the moderating effect of self-control management to improve employee performance. The model incorporates excessive social use at work, excessive hedonic use at work, and excessive cognitive use at work as a stressor, social media exhaustion as a mediator to reduce employee performance, and self-control management as a moderator to significantly improve employee job performance which is the dependent variable (see Figure 1). The context of this study precisely identifies the process through which excessive social, hedonic and cognitive use at work impairs each employee’s capacity for performance.

## 4 Research methodology

### 4.1 Sampling procedures and measurements

The purpose of this study was to examine how social media usage affects university academic staff members’ job performance. This study targeted academic staff performance because they are the main factor that determines a university’s ranking; nonetheless, it is evident that staff members are not performing to the best of their abilities, which is the real reason why the institution does not have a high ranking (Iqbal et al., 2017). Using social networking sites (SNSs) during working hours may have an impact on academicians’ performance. This could result in poor academic achievement, which would prevent universities from moving up the university ranking.

The Along with the goal of the research, the measurement items of the questionnaires were disseminated via an internet platform utilizing an email. The tested scales that we used in this investigation were modified from earlier works. To meet the setting of the study, the questionnaire’s phrasing was changed. The five items of excessive social use at work were developed by Ali-Hassan et al. (2015) to measure particular constructs. The three-item and four-item excessive hedonic and cognitive use at work scores were modified by Ali-Hassan et al. (2015). The social media exhaustion measurements were modified by Ayyagari (2012). The four performance indicators were taken from Janssen and Van Yperen (2004). A total of 16-item self-control and the self-management measure were modified (Mezo, 2009). A Likert scale with five possible values was used to score each item.

As control variables, several demographic factors, such as gender, age, education, industry type, and the frequency and duration of social media use, were included in the constructs in the proposed research model. To gather the data for this investigation, an online survey was used. Five of Malaysia’s major universities provided the necessary sample. Therefore, the target participants were university faculty members who had some background in using social media in businesses 391 individuals participated in total. Our research is deeply entrenched in the field of quantitative research. Quantitative research is well-suited to determining the values of variables, supporting robust statistical analysis, and allowing for a methodical investigation of correlations within a specific population. To achieve study research goals, this study used a cross-sectional research strategy, which is distinguished by its concentration on collecting data from one group or demographic at a single time. The use of a cross-sectional methodology was chosen since it enabled us to obtain an overview of the phenomenon under inquiry while also successfully assessing variations and linkages within our target group. The sampling approach adopted for the present research was purposeful sampling, owing to its connection with the research’s unique objectives and emphasis. This method of non-random sampling entails a deliberate choice of respondents who have the necessary traits or knowledge about the study’s issue. The potential negative consequences of excessive social media use on workers’ ability to execute their jobs were investigated in this study. We adopted the purposive sampling method to provide each University Employee in Malaysia an equal chance of being selected even if we do not have the exact list of employees that may be our respondents. The targeted respondents were contacted by email once the questionnaire was generated using Google Forms (Sekaran, 2003). We had to rely on online data collection because there weren’t many options for physical data collection during a pandemic. By leveraging online methods for data collecting, we can guarantee both the respondents’ security and timely data gathering.

### 4.2 Data analysis

#### 4.2.1 Demographic characteristics

The demographic characteristics of the respondents (see Table 1), identified in this study are as follows: valid responses received 391, where 53.2% (209) were males and 46.8% (184) were females in the present field. All the identified respondents of the study were adults, where most of the respondents were found within the age groups of 41–50 were 126 (32.1%), 25–30 was 122 (31%), 31–40 were 113 (28.8%), respectively, followed by age groups 51 years and above were 32 (8.1%). While 240 (61.4%) are Malay, Chinese 138 (35.3%), Indian 5 (1.3%), followed by 11 others. Based on education, 295 of the total respondents are Ph.D. (75.4%), 47 are master’s degrees (12%), and 48 are Bachelor’s (12.3%). Based on job experience 128 respondents are having less than 5 years (32.7%), Less than 10 years are 107 (27.4%), less than 15 years are 86 (22%), and more than 15 years of experience are 70 (17.9%). Therefore, such a profile of respondents indicates that they are mature, educated, and experienced profile, and have a deeper understanding of the topic under investigation.

**TABLE 1:** Demographics.

Demographic	Frequency rate	Percentage
**Sex**		
Male	209	53%
Female	184	46%
**Age group**		
25–30	122	31%
31–40	113	28.80%
41–50	126	32.10%
51–above	32	8.10%
**Race**		
Malay	240	61.40%
Chinese	138	35.30%
Indian	5	1.30%
**Education**		
PhD	295	75.40%
Masters	47	12%
Bachelor’s	48	12.30%
**Experience**		
Less than 5 years	128	32.7%
Less than 10 years	107	27.4%
Less than 15 years	86	22%
More than 15 years	70	17.9%

## 5 Results

R-squared, is a commonly used tool in statistical evaluation to demonstrate how much of the variance in the dependent variable can be determined by each of the independent variables. This study purposefully employs SmartPLS for analysis in this study rather than R-squared, taking into account because SmartPLS is a structural equation modeling (SEM) approach that works especially well with low sample sizes and sophisticated models that contain latent variables. Both formative and reflective measuring models are supported. The study chose this approach to better fit the intricate details of study model. SmartPLS is well-known for being resilient to unconventional data, which makes it a good fit in scenarios where the R-squared assumptions of conventional linear regression models do not hold true. Considering the sensitive nature of this study data, this was highly important. A key component of SmartPLS is predictive relevance, whereas R-squared is mainly concerned with interpreting variance. This is in line with the study’s goals, which included forecasting and offering recommendations in addition to elucidating the correlations between the variables. Although it is customary to utilize R-squared as a coefficient of determination, the choice to stray from this method and use SmartPLS was reached after taking the particulars and goals of the study into account. Methodological fit, resilience, predictive relevance, adaptability, and the capacity to extract practical insights were prioritized.

SmartPLS 3.3.2 was employed in this study’s analysis of the data gathered. To analyze the data, PLS-SEM has been divided into two steps (Hair et al., 2017). The reliability and validity of the discovered constructs are evaluated in the primary phase using the PLS method. The following stage involves evaluating the outcomes using a structural model and bootstrapping.

## 6 Assessment of the measurement model

Using structural equation modeling with partial least squares, confirmatory factor analysis was performed (PLS-SEM). According to this research, the SmartPLS technique was used to examine a fictitious framework from a prediction perspective.

In the initial stage factor loadings of identified indicators were estimated, followed by the Cronbach alpha value, and at last the average variance was examined of the constructs’ reliability and validity (AVE). The average Cronbach alpha for excessive cognitive use at work is 0.790, which is considered good for testing validity and reliability. The reliability of Cronbach’s alpha for excessive hedonic use at work is 0.811. For excessive social use at work, Cronbach alpha reliability is 0.745. The social media exhaustion scale’s Cronbach alpha reliability is 0.864. Cronbach alpha reliability for self-control management is 0.857. The job performance scale’s Cronbach alpha value is 0.796. All the values mentioned here show no reliability issue because identified constructs values are more than the threshold values.

The next step is to complete confirmatory factor analysis (CFA) was executed by applying structural equation modeling based on partial least squares (PLS-SEM). The SmartPLS practice was applied by Koay et al. (2020), Leung et al. (2020), and Cheung et al. (2021) because their research examined a hypothetical relationship framework from a prediction view.

All the mentioned ways to assess construct reliability and validity are mentioned (Hair et al., 2017). All the factor loading of all indicators was examined in an initial phase, later on, the Cronbach alpha value, and lastly the average variance extracted (AVE) was extracted (Henseler et al., 2009).

### 6.1 Construct reliability and validity

All the items of variables were embraced in the model, however after the evaluation process, items having factor loading less than 0.50 were removed (Hair et al., 2011, 2013). The recommended threshold values for outer loading are 0.60 or above, and composite reliability (CR) and values for Cronbach’s alpha (CA) should be above 0.70. To obtain the convergent validity with the AVE must be > 0.50. This study results met all the identified threshold values and depicts reliable constructs in Table 2.

**TABLE 2:** Construct reliability and validity.

	Cronbach’s alpha	rho_A	Composite reliability	Average variance extracted (AVE)
ECUW	0.790	0.796	0.866	0.621
EHUW	0.811	0.814	0.888	0.726
ESUW	0.745	0.748	0.831	0.498
JP	0.796	0.798	0.869	0.626
SMEx	0.864	0.865	0.902	0.647

### 6.2 Discriminant validity

Discriminant validity was assessed by the Heterotrait-Monotrait Ratio (HTMT) in methodology which is regarded as a significant method to examine the value of discriminant validity. Literature used the regression analysis method to evaluate the effect of the exogenous variables excessive cognitive use at work, excessive hedonic use at work, and excessive social use at work on employee job performance, with social media exhaustion serving as a mediator. The results of the study demonstrated that, when social media exhaustion taking into account, all the exogenous variables significantly affect the job performance of employees, except for excessive hedonic use at work. As recommended by Hair et al. (2017), SmartPLS was used to carry out the bootstrapping technique with 5,000 resamples. All the values show that there is no discriminant validity issue (see Table 3).

**TABLE 3:** Discriminant validity.

Discriminant validity (HTMT)					
	ECUW	EHUW	ESUW	JP	SMEx
ECUW					
EHUW	0.394				
ESUW	0.611	0.684			
JP	0.643	0.360	0.640		
SMEx	0.769	0.484	0.766	0.824	

### 6.3 Assessment of structural model

**FIGURE 2:**
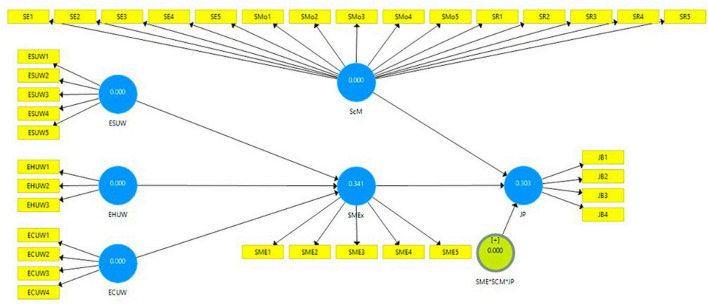
Blindfolding. *Is used in analyzing the moderation effect of ScM between SME and JP.

**FIGURE 3:**
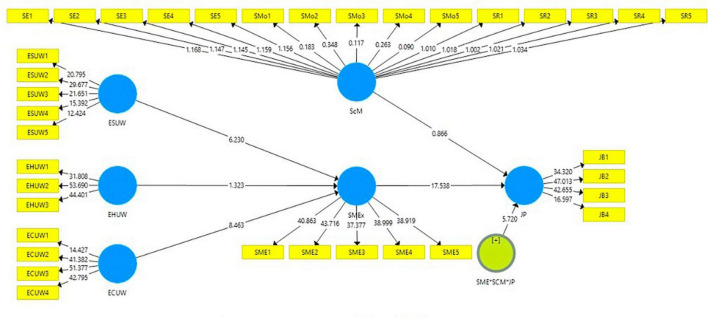
Structural model assessment. *Is used in analyzing the moderation effect of ScM between SME and JP.

The value of Q2 is 0.299, indicating the predictive significance of 30.3% shown in Figure 2 for job performance as a dependent variable. However, the *R*^2^ value for the mediation value is 0.341, indicating that 34.1% predict task distraction affecting job performance, and the value of Q2 is 0.534, indicating that task distraction has a predictive relevance. The structural model assessment depicted in Figure 3 was used in this work to examine the theory after the measurement model generated the necessary results. Analysis of the statistical significance, T-value, and effect size of the route coefficient was done using the bootstrapping of 5,000 samples (Jeon et al., 2019).

Meanwhile, multiple regression has been employed to examine the effects of the independent variable’s excessive social use at work, excessive hedonic use at work, and excessive cognitive use at work overload leading to social media exhaustion and decrease employee job performance. The findings showed the usage of social networking sites and apps increases employee interest in excessive information, cognitive and hedonic use at work increase and leads to social media exhaustion and decrease job performance.

This study depicted that employees while using excessive social media exhaust them to perform their tasks and decrease their job performance. Earlier studies also retrieved social media effective in reducing the employee performance output (Zhan et al., 2016), whereas SSO was also found effective in influencing employee efficiency on the job (Zhang et al., 2015; Liu et al., 2017; Al Hammadi and Hussain, 2018; Lee and Lee, 2018; Sherman et al., 2018; Javed et al., 2019; Yu et al., 2022). So, this study found the core related issues of excessive social media use (ESMU), excessive hedonic use and excessive cognitive use at work, social media exhaustion, and employee job performance (see Table 4). In this study, except for excessive hedonic use at work all of the factors were found significant to influence job performance.

**TABLE 4:** Structural model assessment.

	Original sample (O)	Sample mean (M)	Standard deviation (STDEV)	T statistics (| O/STDEV|)	*P*-values
ECUW > SMEx	0.443	0.443	0.052	8.592	0.000
EHUW > SMEx	0.066	0.066	0.049	1.327	0.092
ESUW > SMEx	0.373	0.374	0.060	6.250	0.000
SME*ScM > JP	0.124	0.158	0.059	2.117	0.017
SMEx > JP	−0.656	−0.655	0.033	19.760	0.000

*Values shows the moderating effect examined.

According to Cohen (2013) general guidelines, values of (f2) between 0.02 and 0.15, between 0.15 and 0.35, and over 0.35 indicate that the exogenous construct has, respectively, a mild, moderate, and substantial effect on the dependent variable. While results below 0.02 suggest that there is no effect, but when (Aguinis et al., 2017) examined earlier research from the previous three decades, and discovered that the average moderating impact is 0.009. In recent research, (Kenny, 2018) proposed 0.005, 0.01, and 0.025 as small, medium, and large effect sizes of moderation, respectively, which represent more practical criteria. The fact that even these figures are optimistic is also highlighted by him. The following formula can be used to compute the magnitude of the moderating effect size (f2):


$$\begin{array}{l} \text{Moderation Effect Size:}\,f^{2}\\ \;=\frac{R_{m\,o\,d\,e\,l\,w\,i\,t\,h\,m\,o\,d\,e\,r\,a\,t\,o\,r}^{2}-R_{m\,o\,d\,e\,l\,w\,i\,t\,h\,o\,u\,t\,m\,o\,d\,e\,r\,a\,t\,o\,r}^{2}}{1-R_{m\,o\,d\,e\,l\,w\,i\,t\,h\,m\,o\,d\,e\,r\,a\,t\,o\,r}^{2}} \end{array}$$

where,


f2=moderation effect size (change in R2 value of the endogenousconstruct due to moderator/interaction term).

Rm⁢o⁢d⁢e⁢l⁢w⁢i⁢t⁢h⁢m⁢o⁢d⁢e⁢r⁢a⁢t⁢o⁢r2=the value of R2 exogenous construct when the interactionterm of the moderator model is included.

Rm⁢o⁢d⁢e⁢l⁢w⁢i⁢t⁢h⁢o⁢u⁢t⁢m⁢o⁢d⁢e⁢r⁢a⁢t⁢o⁢r2=the value of R2 exogenous construct when the interactionterm of the moderator model is excluded.

$$\begin{array}{l} f^{2}=\frac{0.519-0.479}{1-0.519},=f^{2}=\frac{0.04}{0.481}\\ f^{2}=0.083 \end{array}$$

According to the Kenny propositions, the value of *f*^2^ was more than the large effect that is 0.025 > it shows that the social media effect has large effect to influence consumer purchase intention (see Table 5).

**TABLE 5:** *F*^2^ values effect.

*F* ^2^	Weak	Moderate	Large
Propositions	0.005 >	0.01 >	0.025 >

### 6.4 Discussion and hypotheses testing

This study’s results proved the significant relationship between excessive cognitive use at work on social media exhaustion. The broad amount of cognitive usage of social networking sites obtained by employees upsurges social media exhaustion and diverts them from the task identified, as depicted values *P*-values (0.000). Result of the cognitive usage are consistent with the Deryakulu and Ursavaş (2014), which shows that social media cognitively usage by sharing ideas, photos, debates etc., distracts employee from identified tasks. Landers and Schmidt (2016) also supported that employees while investing his time on exploring ideas and information during office hours divert from actual task and gains exhaustion.

Hedonic use at work also causes dysfunctional consequences, while excessive usage of social media diverts employees from identified tasks. Excessive hedonic use at work *P*-values (0.092) indicates that it is not significant. Result of this factor are consistent with Luqman et al. (2017), which shows that usage of social media for fun at workplace causes exhaustion among employees and significantly distract them from their identified tasks. A study by Benson et al. (2019) also found a connection between the usage of SNS hedonically among students with reduced performance in the classroom.

Excessive social use at work *P*-values (0.000) shows that the relationship between excessive social use at work and social media exhaustion is significant. Excessive usage of social media distracts users and reduces the level of employee performance. With the usage of social networking sites, employees’ social media exhaustion increases and decreases the level of employee performance. *P*-values (0.000) show a significant relationship in reducing employee performance. The results supports the earlier studies which demonstrate that overuse of social media encourage users of constant checking of social media at the workplace, which leads to compulsive behaviors and distract from actual job (Yu et al., 2018). Another study also found that SNS social users during office hours lacks effective self-control, which interferes with daily activities in the home, workplace, schools, and businesses and reduce their performance (Cao and Sun, 2018).

As the usage of social networking sites exhausts employees from the usage of social media, with the effective usage of self-control management employee performance can be significantly improved. The *P*-values (0.017) show a significant relationship between self-control management and job performance. Earlier studies also revealed that self-management is essential to keep track of one’s behavior of getting distracted by excessive SNS use during office timings to improve performance (Wheelock et al., 2015). Usually, a self-managed person makes strategies for themselves and works hard to achieve those objectives by improving their performance (Javed et al., 2019; Majid et al., 2019). Self-management considered as a moderating variable between social media exhaustion and employees’ performance based on the hypothesis that self-managed employees perform better, it significantly moderate the association.

Due to the rapid growth of technology and knowledge, there is also a risk of social media tiredness (Lee S. L. et al., 2016; Moughal et al., 2023b). Additionally, since the quantity of social media users grows tremendously, information is shared quickly on these platforms. Users of social media may experience overwhelming amounts of information due to cognitive overload during their office hours which negatively impacts employee performance (Eliyana et al., 2020). The current study shows a link between cognitive use at work and social media tiredness that is in favor of communication overload which reduces employee performance. The impact of cognitive use at work on social media weariness is due to the fact that “social media consumes employees too much time and has the greatest magnitude of reducing employee performance.” During office hours, usage of social media exhausts employees and distracts them from their assigned tasks. Social networking sites are one of the communication tools that connect employees inside organizations with others to communicate (Ou and Davison, 2011), however the usage of social media for personal or social use for enjoyment will distract employees and reduce their performance. According to Karr-Wisniewski and Lu (2010) and Eliyana et al. (2020), when employees get communicated with others via social networking sites, they frequently stop what they are doing and respond right away. An individual will require a few moments to resume stopped work operations after handling communication disruptions (Karr-Wisniewski and Lu, 2010).

The study’s findings demonstrate that excessive usage of social networking sites has a detrimental effect on work performance. Employee excessive usage of social sites is the root of the reduction in performance. Employees who receive too much interaction lose connection with their assigned job tasks and feel unproductive because they socially interact with others online, and their performance suffers as a result. This study revealed the possible solution which can be helpful to control their social media exhaustion and improve their performance with the help of self-control management. Self-control management significantly moderates the relationship between social media exhaustion and job performance among academic employees. Employees who communicate online too much, frequently make mistakes and mislead from assigned job duties, with the identified factor self-control management employees can control their usage of social media and improved performance. Employees that get excessive communication have a tendency to just pay attention to particular information and self-control and disregard other information to improve their job performance.

### 6.5 Theoretical implications

The study covered theoretical ramifications; firstly, rather than focusing on the positive side, the research can expand the body of self-control management literature to improve employee performance, which is largely ignored in the conceptualizations; drivers, and effects of SNS usage (Tarafdar et al., 2020a). To highlight the behavioral outcome as a coping mechanism for stressful situations, we looked at the antecedents of low academic performance as SNS stressors. The present research specifically analyses the potential negative impacts of problematic social networking site (SNS) usage on academic performance and broadens the theoretical and empirical understanding of the etiology of problematic SNS use. This study brought attention to the SNS stressors by using the SSO framework. It helped to provide a clearer and more thorough knowledge of inappropriate SNS use, which results in stress in the shape of low job performance. It is important to consider the causes of low academic performance, particularly when they are brought on by an unbalanced flow of SNS usage. Furthermore, to some extent, this study adds to the body of social networking sites’ self-control management and SSO theory literature. In particular, it reveals that SSO philosophy can be employed to pinpoint important factors like excessive social use at work, excessive cognitive use at work, and excessive hedonic use at work in addition to being an appropriate explanation for inconsistent usage of SNS. In comparison to planned behavior, SSO theory is more effective in explaining less rational behavior. This makes a significant contribution to the body of literature on the usage of SNSs during office hours to improve job performance. Thirdly, this research supports not only the development of SSO theory but also the comprehension of self-control management to improve employee performance.

This is an important contribution to SNSs use literature that has mainly relied on planned behavior research (Shin and Hall, 2011). Thirdly, in addition to the importance of SSO theory, this research also subsidizes developing an understanding of discontinuance intention and the underlying mechanism involved. We considered different discontinuance intentions behavioral patterns such as suspending the usage, rationalizing the usage, and permanently leaving the use of SNS which is consistent with the prevailing scholars in IS (Hwang et al., 2019). This study considered factors of SNS i.e., excessive social, cognitive, and hedonic use at work, which are in line with views of distracting employees from their assigned jobs (Hwang et al., 2019), and the usage of social media exhaust employees and distract employees.

Additionally, the moderating role of self-control management enhanced the employee to refrain from using SNSs during office hours who experience poor academic performance as a result of SNSs stresses, it is also considered a significant contribution to the existing literature. In other words, it suggests that employees during office timings reduce SNS usage as a coping mechanism to avoid stressful circumstances in the future (Zhan et al., 2016). Ultimately, this study contributed by evaluating all three kinds of stressors (excessive social use at work, excessive hedonic use at work, and excessive cognitive use at work) as social media-related stressors and examining their impact on academic performance.

The aforementioned stressor is prevalent among employees and is covered in the social media aspect (Luqman et al., 2017). These three types are hardly ever discussed in relation to academic performance, although results show that they are all significant sources of SNSs linked to stressors that have an impact on employees’ academic performance. Additionally, our study demonstrates the significance of stimulus features in impacting academic performance with the use of SSO frameworks, such as social media exhaustion can be released with effective usage of self-control management. This discovery contributes to SNS-related research and exposes the negative effects of SNSs use by demonstrating cognitive attention spurred by SNS stimuli, which has an impact on academic performance. Particularly, our research demonstrates the clear link between SNSs affecting employee job performance and deviating their attention.

### 6.6 Practical implications

To keep an eye on the negative impacts of SNSs use, this research also makes several practical recommendations for SNSs users and educational institutions. First, our data show that an excessive usage pattern of SNSs is to blame for the link between stress and low academic performance. Making it simpler to manage and monitor their usage can reduce the stress brought on by SNSs overuse and improve performance. Second, this study has significant implications for organizations that want to increase employee performance by managing their excessive SNSs usage during office hours. Organizations can host seminars to explain why using SNSs excessively is risky, why employees should be careful and avoid using SNSs during office timings, and how to stop its negative consequences. SNS usage can be reduced even slightly to benefit organizations by improving employee performance.

It is crucial to highlight the need for effective instruction regarding the harmful implications of SNSs usage and adaptation in the workplace and educational institutes as well. Ultimately, this study highlights the importance of a logical point of view, which is a relatively new paradigm of SNSs stress reduction by self-control management. The available empirical findings support the current literature, which suggests that employee should moderate their behavior while using SNS to avoid unpleasant emotions and the possibility that they may negatively impact academic performance.

### 6.7 Limitations and future recommendations

The approach this study used to collect data was online, online lagged survey data do not allow for the assessment of connection since they may be impacted by distracting variables and do not clearly indicate the direction of effect between the examined constructs. Different approach for data collection should be used in future studies to get specific direction and actual relationship between the variables. Furthermore, this study was limited to data collection of universities employees of Malaysia, future studies can target other organizations’ employees as well to confirm the generalizability. Furthermore, the effects of stressors excessive social use, excessive hedonic use, and excessive cognitive use at work, and other factors like information overload, and system feature overload can be observed in other aspects of life, such as the workplace. The “tipping point” at which individuals begin to experience stress using SNSs has to be rigorously investigated in the future.

Furthermore, while we have utilized gender as well as age as controls in Malaysian context, future studies should examine these control factors in other part of world. Other characteristics that are linked to excessive SNSs usage, such as drinking and smoking, which affect the employees’ academic success can also be used. These elements may aid in identifying additional effects associated with subpar academic achievement. The framework suggested in this study should be expanded in subsequent investigations. The indirect impact of social media exhaustion can also be examined in a later study, which can also include additional variables pertaining to beliefs and actions in linked research domains.

## Data availability statement

The raw data supporting the conclusions of this article will be made available by the authors, without undue reservation.

## Ethics statement

Ethical review and approval was not required for the study on human participants in accordance with the local legislation and institutional requirements. Written informed consent from the patients/participants or patients/participants legal guardian/next of kin was not required to participate in this study in accordance with the national legislation and the institutional requirements.

## Author contributions

WM: Conceptualization, Data curation, Formal analysis, Methodology, Software, Validation, Writing – original draft. SN: Funding acquisition, Supervision, Writing – review & editing. RS: Funding acquisition, Resources, Supervision, Validation, Writing – review & editing. HA: Formal analysis, Software, Validation, Writing – original draft.
